# What do we need to move enzybiotic bioinformatics forward?

**DOI:** 10.3389/fmicb.2024.1474633

**Published:** 2024-09-05

**Authors:** Sophia Bałdysz, Krystyna Da̧browska, Jakub Barylski

**Affiliations:** ^1^Department of Molecular Virology, Institute of Experimental Biology, Adam Mickiewicz University, Poznań, Poland; ^2^Faculty of Medicine, Wroclaw Institute of Science and Technology, Wrocław, Poland

**Keywords:** enzybiotic, bioinformatics, forward, consortium, lysins

## Abstract

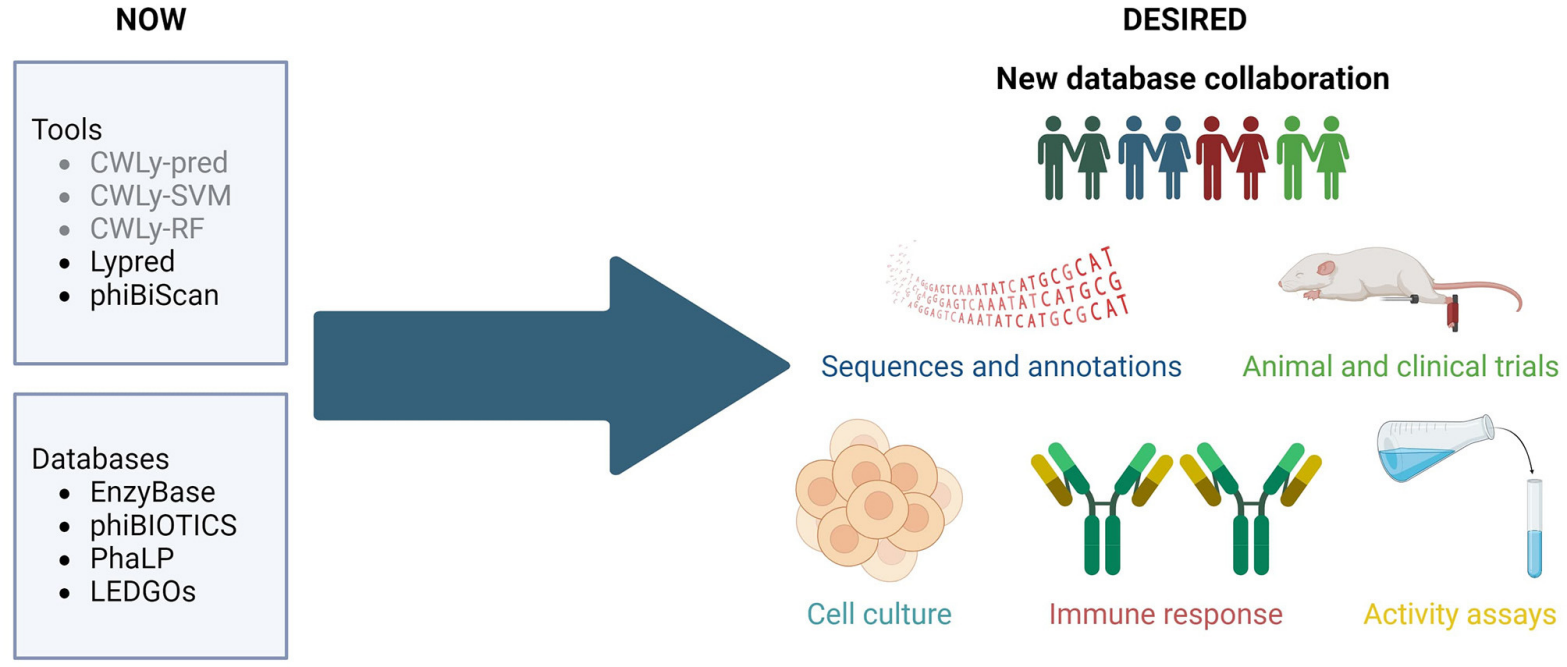

## Highlights

- Lytic enzymes are a promising alternative to treating antibiotic-resistant bacteria.
- Many tools and databases developed to study lysins are no longer maintained or outdated.
- This paper reviews the current state of endolysin computational methods.
- There is an opportunity for the scientific community to develop a tailored database for these proteins with coherent ontology.

In the age of increasing numbers of infections caused by antibiotic-resistant bacteria alternative strategies for combating these superbugs are in high demand. One of the most promising approaches involves the use of lytic enzymes, or simply enzybiotics such as autolysins, bacteriocins, endolysins, and virion-associated lysins, as well as biofilm degrading depolymerases. The effectiveness of such proteins has been proven in numerous *in vitro* studies, animal models, and several clinical trials (Murray et al., 2021; Schmelcher and Loessner, 2021; Liu et al., 2023). Unfortunately, enzybiotics targeting many important pathogens are still unavailable and identification of novel therapeutic proteins through traditional wet-lab methods is time-consuming and expensive. Publicly available databases provide access to millions of metagenomic sequences that could serve as a virtually inexhaustible source of novel lytic enzymes. However, identification of enzybiotic-coding sequences and matching them with susceptible bacteria still remains the major problem.

In previous years several bioinformatic tools have been developed for searching for bacteriolytic proteins. These included machine-learning based classifiers, designed to distinguish between lytic and non-lytic proteins based on the frequencies of amino acids within the proteins, as well as their order in the sequence (Lypred, CWLy-SVM, CWLy-pred and CWLy-RF) (Chen et al., 2016; Meng et al., 2020a,b; Jiao et al., 2021). Unfortunately, all of the tools used a very similar small, unbalanced, and barely curated collection of sequences to construct training and testing datasets. Additionally, one may wonder if authors of some of these tools (Chen et al., 2016) had enzymological knowledge required to critically evaluate bioinformatic results since they referred to lytic proteins as “lyases”. Importantly, Lypred has not been updated since its release and the other tools are not available.

Currently, the only accessible tool is phiBiScan, which uses 16 models (profile hidden Markov models) representing conserved lysin-related domains to search for lytic proteins. Although versions of these models are regularly updated (the current version of this tool uses profiles from Pfam 35.0), the list of lysin-related domains has not been revised since its release in 2013 (Hojckova et al., 2013). It seems unlikely that just 16 domains reflect the entire diversity of lytic proteins observed in nature (Fernández-Ruiz et al., 2018; Bałdysz et al., 2024).

All of these examples demonstrate that although bioinformatic lytic protein detection tools have been developed, their use is restricted mainly to homologs of known proteins, and the repertoire of well characterized enzybiotics is rather limited. More importantly, it is difficult to assess the effectiveness of programs developed to identify enzybiotics because we simply do not have a representative test set of validated enzybiotic sequences.

The databases published up to date (EnzyBase, phiBIOTICS, PhaLP, and LEDGOs) (Wu et al., 2012; Hojckova et al., 2013; Criel et al., 2021; Mitchell et al., 2021) are either too small (e.g., hold < 1,000 enzymes) and/or rely heavily on *in silico* annotation instead of experimental information. They are also taxonomically biased—only a handful of protein groups (e.g., against staphylococci) are well represented in these databases. What's more discouraging, the majority of the included sequences have been selected based merely on similarity but the real range of their activity has not been validated by wet-lab methods. Additionally, most lysin databases have not been updated in many years and some are no longer available. Obviously, the lack of large, well annotated, enzybiotic databases is particularly detrimental to the development of machine-learning tools, because these require comprehensive well-balanced training and test sets. The same can be concluded about the inconsistent, and poorly standardized metadata, which does not follow any formal ontology and often fails to track current taxonomy. Hence, although such lysin identification tools are desperately needed in the scientific market, they do not reach broader researchers' audiences and do not gain recognition.

The research community needs a representative and consistent database containing enzybiotic sequences, along with accurate, detailed annotations, wet-lab confirmation of the activity of the protein, and, if available, results from animal tests or clinical trials, along with other relevant information, like safety for human cells or immunogenicity.

We firmly believe that such a database shouldn't result from the work of one specialized group, to avoid bias from this group's specific scientific background. Instead, it should be a collective work of the larger community. Such an approach will ensure that the structure of the new database and the information stored within will cater for the needs of diverse groups, including enzymologists, bioinformaticians, machine-learning specialists, medical professionals or biotechnology and pharmaceutical companies. We firmly believe that collaboration between different laboratories, regular maintenance of tools and databases, as well as exploration of novel *in silico* methods may prompt flourishing of enzybiotics studies leading to numerous new breakthroughs. Therefore, we call for the creation of a consortium that will prepare a tailored database, guarantee its coherent, formalized ontology and sequence nomenclature, gather scattered sequences and integrate biochemical, molecular and evolutionary information, like domains and families. Current boom in language processing tools may also be a unique opportunity to include literature information in a consistent manner, while under careful supervision of human curators.
